# Free fatty acid biosynthesis precursors are involved in pollen–stigma interactions in *Brassica*

**DOI:** 10.1093/hr/uhaf147

**Published:** 2025-06-11

**Authors:** Hongtao Qin, Kumar Abhinandan, Min Wang, Huan Chen, Xue Zhang, Lanlan Li, Zhengwenyang Zhou, Suhui Wang, Chaoning Zhao, Wendi Mu, Yongxue Yuan, Ying Wu, Yuhua Li, Marcus A Samuel, Xingguo Lan

**Affiliations:** Key Laboratory of Saline-Alkali Vegetation Ecology Restoration, Ministry of Education, College of Life Sciences, Northeast Forestry University, No. 26 Hexing Road, Xiangfang District, Harbin, Heilongjiang 150040, China; Department of Biological Sciences, University of Calgary, 2500 University Drive N.W., Calgary, Alberta T2N 1N4, Canada; Key Laboratory of Saline-Alkali Vegetation Ecology Restoration, Ministry of Education, College of Life Sciences, Northeast Forestry University, No. 26 Hexing Road, Xiangfang District, Harbin, Heilongjiang 150040, China; Key Laboratory of Saline-Alkali Vegetation Ecology Restoration, Ministry of Education, College of Life Sciences, Northeast Forestry University, No. 26 Hexing Road, Xiangfang District, Harbin, Heilongjiang 150040, China; Key Laboratory of Saline-Alkali Vegetation Ecology Restoration, Ministry of Education, College of Life Sciences, Northeast Forestry University, No. 26 Hexing Road, Xiangfang District, Harbin, Heilongjiang 150040, China; Key Laboratory of Saline-Alkali Vegetation Ecology Restoration, Ministry of Education, College of Life Sciences, Northeast Forestry University, No. 26 Hexing Road, Xiangfang District, Harbin, Heilongjiang 150040, China; Key Laboratory of Saline-Alkali Vegetation Ecology Restoration, Ministry of Education, College of Life Sciences, Northeast Forestry University, No. 26 Hexing Road, Xiangfang District, Harbin, Heilongjiang 150040, China; Key Laboratory of Saline-Alkali Vegetation Ecology Restoration, Ministry of Education, College of Life Sciences, Northeast Forestry University, No. 26 Hexing Road, Xiangfang District, Harbin, Heilongjiang 150040, China; Key Laboratory of Saline-Alkali Vegetation Ecology Restoration, Ministry of Education, College of Life Sciences, Northeast Forestry University, No. 26 Hexing Road, Xiangfang District, Harbin, Heilongjiang 150040, China; Key Laboratory of Saline-Alkali Vegetation Ecology Restoration, Ministry of Education, College of Life Sciences, Northeast Forestry University, No. 26 Hexing Road, Xiangfang District, Harbin, Heilongjiang 150040, China; Key Laboratory of Saline-Alkali Vegetation Ecology Restoration, Ministry of Education, College of Life Sciences, Northeast Forestry University, No. 26 Hexing Road, Xiangfang District, Harbin, Heilongjiang 150040, China; Key Laboratory of Saline-Alkali Vegetation Ecology Restoration, Ministry of Education, College of Life Sciences, Northeast Forestry University, No. 26 Hexing Road, Xiangfang District, Harbin, Heilongjiang 150040, China; Key Laboratory of Saline-Alkali Vegetation Ecology Restoration, Ministry of Education, College of Life Sciences, Northeast Forestry University, No. 26 Hexing Road, Xiangfang District, Harbin, Heilongjiang 150040, China; Department of Biological Sciences, University of Calgary, 2500 University Drive N.W., Calgary, Alberta T2N 1N4, Canada; Key Laboratory of Saline-Alkali Vegetation Ecology Restoration, Ministry of Education, College of Life Sciences, Northeast Forestry University, No. 26 Hexing Road, Xiangfang District, Harbin, Heilongjiang 150040, China

## Abstract

Self-incompatibility (SI) is a complex molecular mechanism in flowering plants that prevents self-fertilization and promotes outcrossing. We conducted metabolome analysis of ornamental kale (*Brassica oleracea* var. *acephala*) pistils following SI and compatible pollination (CP), revealing significant alterations in lipid metabolism, particularly the accumulation of free fatty acid (FFA) metabolites during CP. Treatment of stigmas with acetyl-CoA and malonyl-CoA, key precursors in fatty acid (FA) synthesis, broke down SI and enhanced CP. Conversely, inhibiting acetyl-CoA carboxylase (ACCase), the rate-limiting enzyme in *de novo* FA synthesis, significantly reduced compatible pollen attachment and tube growth, highlighting the critical role of FA metabolism in mediating pollination success. We identified a novel interaction between the FERONIA (BoFER) receptor kinase and the biotin carboxyl carrier protein 1 (BoBCCP1), a subunit of the ACCase complex. Suppressing the expression of *BoBCCP1* in the stigma reduced CP response, suggesting that the FER-BCCP1 module may play a crucial role in regulating FA biosynthesis and determining the outcome of pollen–stigma interactions. Our findings provide new insights into the identification of key metabolic pathways and signaling modules controlling pollen-stigma interactions, and offer a valuable resource for the targeted improvement of *Brassica* crop breeding.

## Introduction

Successful pollination in flowering plants relies on precise communication between pollen and pistil, mediated by complex signaling pathways that determine whether an arriving pollen grain will be accepted or rejected. Many plants employ self-incompatibility (SI) system to recognize and block ‘self’ pollen, avoiding inbreeding depression by enforcing outcrossing. In self-incompatible Brassicaceae family including *Arabidopsis* and *Brassica* spp., the SI response is a well-studied example of these complex signaling pathways [1–5].

Recent research has identified additional molecular candidates and mechanisms of novel receptor modules such as S-locus receptor kinase- FERONIA (SRK-FER) that contribute to the SI response, which suggests a more complex receptor–ligand landscape [6, 7]. Additionally, competitive binding between pollen coat protein B-class peptides (PCP-Bs) and stigmatic RALF peptides highlights a dynamic interplay of signals at the pollen–stigma interface [8, 9]. Furthermore, the role of reactive oxygen species (ROS) in regulating pollination success suggests involvement of other metabolic pathways that may interact with the SRK-FER and PCP-Bs/RALF signaling pathways [6, 7, 10]. These new findings have challenged our understanding of the SI response and have opened new avenues for investigating the diverse metabolic and signaling pathways involved in successful pollination in Brassicaceae. In particular, the role of ROS highlights the potential importance of metabolic pathways in regulating pollination success.

Fatty acids (FAs) and their derivatives, particularly free fatty acids (FFAs), are essential metabolites that serve as important energy sources and signaling molecules in plants [11, 12]. The synthesis of FAs begins with the conversion of acetyl-CoA to malonyl-CoA by the enzyme acetyl-CoA carboxylase (ACCase), which is the rate-limiting step in *de novo* FA synthesis [13, 14]. Malonyl-CoA is then used by fatty acid synthases (FAS) to produce long-chain FAs, which can be further elongated to very long chain FAs (VLCFAs) or released as FFAs [15]. These FAs serve as essential building blocks for membrane lipids, which are crucial for plant growth and development [16, 17]. In particular, FAs and their derivatives play a critical role in regulating anther and pollen development, as the elongation of pollen tubes requires FA and membrane lipid synthesis [18]. Consequently, disruption of lipid metabolism can lead to male infertility [19], highlighting the importance of FA homeostasis in reproductive success.

During compatible pollination (CP), the germination of a pollen on the stigma is metabolically a very demanding process requiring a continuous supply of building blocks that will help the pollen tube to grow culminating in fertilization. Building upon the critical role of FAs in pollen development and male fertility, we hypothesized that FA metabolism in the stigma might also play a crucial role in regulating pollen–pistil interactions during pollination. In this study, we performed metabolome analyses of ornamental kale pistils at different time points following self-incompatible and compatible pollination. Our results demonstrate that the synthesis of FFAs is essential during the early stages of pollination and that FFAs are crucial for CP and can break down SI. We further discovered that Biotin carboxyl carrier protein 1 (BoBCCP1), which encodes the main subunit of ACCase, interacts with the kinase domain of the FERONIA (BoFER) receptor. This interaction may lead to the deactivation of ACCase, which is responsible for converting acetyl-CoA to malonyl-CoA, a key step in FFA synthesis. Consequently, the FER-BCCP1 interaction may limit the supply of FFAs necessary for the CP response.

## Results

### Identification of differential metabolites following self-incompatible and compatible pollination response

**Figure 1:**
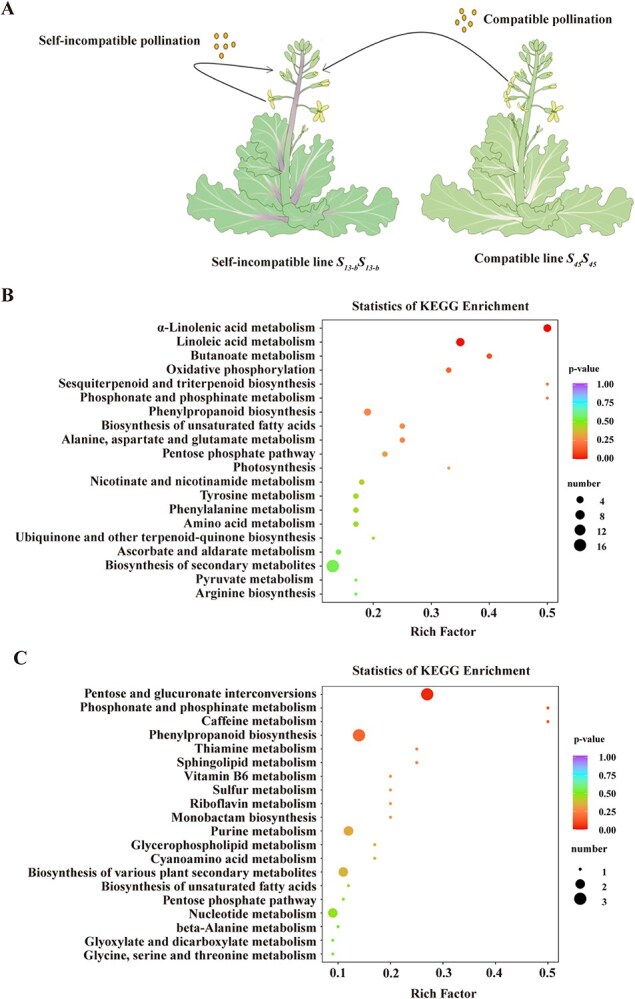
KEGG pathway enrichment analysis of DMs following pollination. **A.** Schematic diagram showing self-incompatible and compatible pollination (CP) in plants. Two *Brassica* plant illustration where pollen transfer is depicted by arrows. Self-incompatible pollination is represented on the left plant, while CP between two different plants is shown on the right. **B.** KEGG enrichment analysis of DMs following CP. The dot plot shows enriched pathways, with dot size representing the number of metabolites and colors indicating the *P*-value. **C.** KEGG enrichment analysis of DMs following self-incompatible pollination. The dot plot shows enriched pathways, with dot size representing the number of metabolites and colors indicating the *P*-value.

To investigate the metabolic changes in pistils during SI and CP responses, we performed widely targeted metabolomic analysis using ultra performance liquid chromatography-mass spectrometry/mass spectrometry (UPLC–MS/MS). Pistils from self-incompatible lines were pollinated with either self-pollen (SI) or cross-pollen (CP) and collected at 30 and 60 min postpollination (Fig. 1A). We identified 1005 metabolites, including phenolic acid, lipids, flavonoids, amino acids and derivatives, alkaloids, organic acids, nucleotides and derivatives, terpenoids, lignans and coumarins, quinones, tannins, and others (Supplementary Fig. S1; Supplementary Table S1). Through filtering based on VIP (variable importance in projection) ≥1 and | Log_2_FC (fold change) | ≥1 differential metabolites (DMs), we identified 159 DMs during pollination; of these, 28 lipid metabolites accounted for the largest proportion of DMs (Supplementary Table S2).

Following SI pollination, 70 DMs (46 upregulated and 24 downregulated) were found in the SI0 versus SI60 comparison group, accounting for the majority; 65 DMs (48 upregulated and 17 downregulated) and 24 DMs (9 upregulated and 15 downregulated) were discovered in the SI0 versus SI30 and SI30 versus SI60 groups, respectively (Supplementary Fig. S2; Supplementary Table S3). Following CP, 83 DMs (60 upregulated and 23 downregulated) were found in the CP0 versus CP60 comparison group, accounting for majority of them; 44 DMs (24 upregulated and 20 downregulated) and 48 DMs (30 upregulated and 18 downregulated) were discovered in the CP0 versus CP30 and CP30 versus CP60 groups, respectively (Supplementary Fig. S2; Supplementary Table S3).

Venn diagram was used to differentiate the common and exclusive metabolites during pollination, there are 93 and 109 DMs following SI and CP, respectively (Supplementary Fig. S3A and B). Following SI, 9 DMs were specific for SI0 versus SI30, 16 DMs were specific for SI0 versus SI60, accounting for the largest proportion, and 2 DMs were specific for SI30 versus SI60 (Supplementary Fig. S3A). Following CP, 5 DMs were specific for CP0 versus CP30; 36 DMs were specific for CP0 versus CP60, accounting for the largest proportion, and 5 DMs were specific for CP30 versus CP60 (Supplementary Fig. S3B). Interestingly, we found three common DMs detected following CP: including 9-Hydroxy-13-oxo-10-octadecenoic acid, p-Coumaryl alcohol, and 9,12,13-TriHOME. A heatmap displaying the expression patterns of three common DMs, which accumulation abundance reaches the highest level following CP60 (Supplementary Fig. S3B and C).

### α-Linolenic acid and linoleic acid metabolic pathways are significantly enriched following CP

Kyoto encyclopedia of genes and genomes (KEGG) enrichment analysis of DMs revealed that α-linolenic acid (*P* = 0.004) and linoleic acid (*P* = 0.012) metabolic pathways were significantly enriched following CP (Fig. 1B; Supplementary Table S4), in contrast to the pentose and glucuronate interconversions (*P* = 0.032) and phosphonate and phosphinate metabolism (*P* = 0.132) pathways that were significantly enriched following SI (Fig. 1C; Supplementary Table S5).

**Figure 2:**
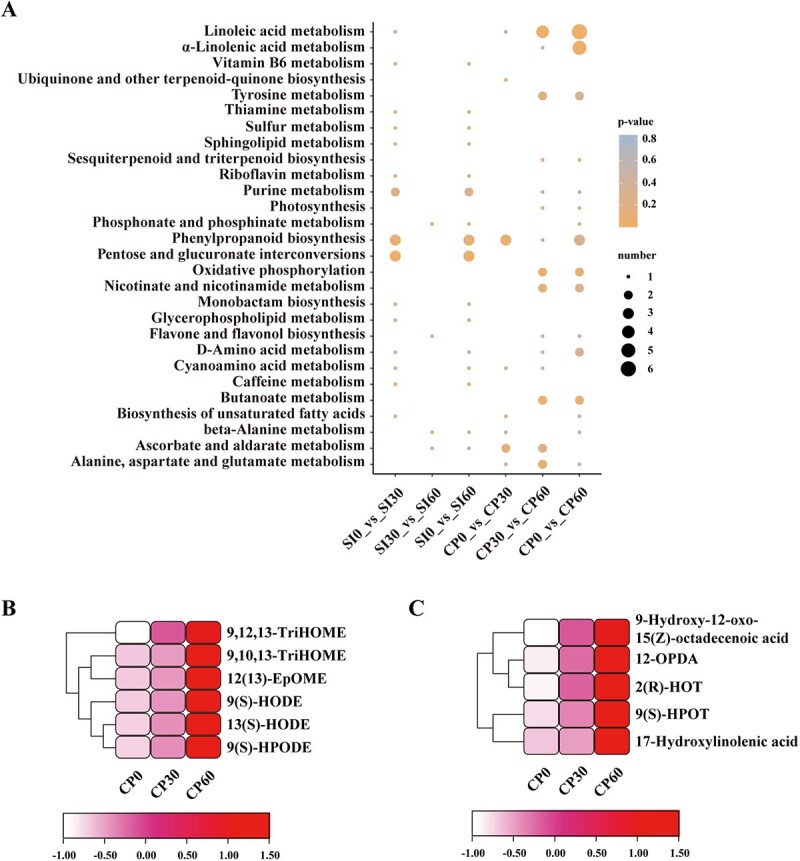
Linoleic acid and α-linolenic acid metabolic pathways are significantly enriched following CP. **A.** KEGG pathway enrichment analysis of DMs across various comparison groups following self-incompatible and compatible pollination. The heatmap-style diagram shows enriched pathways on the y-axis and different comparison groups on the x-axis. Circle symbols represent metabolomics data (DMs). The color gradient from blue to orange indicates increasing statistical significance (lower *P*-value), and the size of the symbols corresponds to the number of DMs involved in each pathway. DMs with *P* ≤ 0.05 were considered significantly enriched in KEGG pathways. **B.** Expression heatmap of DMs from the linoleic acid and (**C**) α-linolenic acid metabolic pathway following CP. The heatmap shows the relative abundance of six metabolites for linoleic acid and five metabolites for α-linolenic acid, respectively, across three different conditions (0, 30, and 60 min).

Building upon the initial KEGG enrichment analysis, we sought to investigate the temporal dynamics of metabolic pathways during early pollination events. To gain further insights into the metabolic pathways involved in early pollination events, we performed a time-resolved KEGG enrichment analysis of DMs following SI and CP. Several metabolic pathways, including vitamin B6, sulfur, thiamine metabolism, sphingolipid, riboflavin, pentose and glucuronate interconversions, monobactam biosynthesis, glycerophospholipid, and caffeine metabolism, were significantly enriched following SI but not CP (Fig. 2A; Supplementary Table S6). In contrast, pathways such as linoleic acid, α-linolenic acid, ubiquinone and other terpenoid-quinone biosynthesis, tyrosine, sesquiterpenoid and triterpenoid biosynthesis, photosynthesis, oxidative phosphorylation, nicotinate and nicotinamide, flavone and flavonol biosynthesis, butanoate, and alanine, aspartate and glutamate metabolism were significantly enriched following CP but not SI (Fig. 2A; Supplementary Table S6). Notably, a higher number of DMs were detected in the linoleic acid and α-linolenic acid metabolic pathways following CP compared to SI (Fig. 2A; Supplementary Table S6).

Our analysis revealed a significant accumulation of several FFAs in the linoleic and linolenic acid metabolic pathways following CP. In the linoleic acid pathway, significant amounts of the downstream metabolites were found accumulating following CP (Fig. 2B): 9,12,13-TriHOME, 9,10,13-TriHOME, 12(13)-EpOME, 9(S)-HODE, 13(S)-HODE, and 9(S)-HPODE. Similarly, the downstream FFAs of the α-linolenic acid pathway significantly accumulated following CP (Fig. 2C): 9-Hydroxy-12-oxo-15(Z)-octadecenoic acid, 12-OPDA, 2(R)-HOT, 9(S)-HPOT, and 17-Hydroxylinolenic acid. The significant accumulation of these FFAs following CP suggests that they may play a crucial role in facilitating pollen hydration and germination during successful pollination.

### Exogenous acetyl-CoA or malonyl-CoA completely break down SI response and increase compatible pollination

Acetyl-CoA is a key precursor in FA biosynthesis, and it is converted to malonyl-CoA by the enzyme ACCase. This reaction is a crucial step in the synthesis of downstream FFAs. Given that CP resulted in accumulation of FFAs that were not observed following SI pollination, we examined whether enhancing FFAs accumulation could lead to alteration in pollen–stigma interaction. To investigate the role of these precursors in pollination, we first examined the effect of treating stigmas with acetyl-CoA on pollen attachment and pollen tube growth following both self-incompatible and compatible pollination.

**Figure 3:**
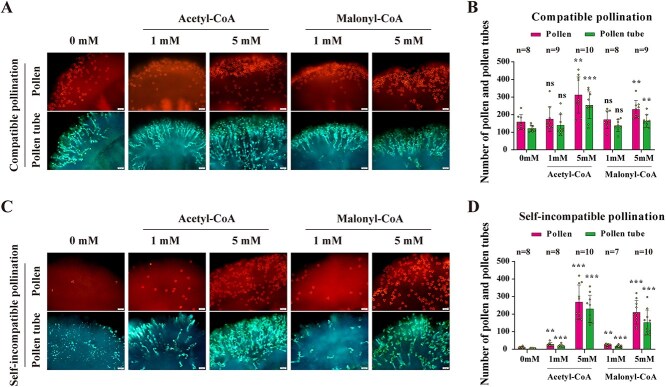
Exogenous acetyl-CoA or malonyl-CoA completely break down SI response and increase compatible pollination. **A.** Fluorescence microscopy images showing pollen and pollen tube growth on *S_13-b_S_13-b_* stigmas following CP. The stigmas were treated with different concentrations (0, 1, and 5 mM) of acetyl-CoA and malonyl-CoA. **B.** Quantification of pollen attachment and pollen tube growth following CP. The data shows a significant increase in both pollen attachment and pollen tube growth with 5 mM acetyl-CoA and malonyl-CoA. **C.** Fluorescence microscopy images showing pollen and pollen tube growth on *S_13-b_S_13-b_* stigmas following self-incompatible pollination. The stigmas were treated with different concentrations (0, 1, and 5 mM) of acetyl-CoA and malonyl-CoA. **D.** Quantification of pollen attachment and pollen tube growth following self-incompatible pollination. The data shows a significant increase in both pollen attachment and pollen tube growth with 1 or 5 mM acetyl-CoA and malonyl-CoA. Statistical significance is indicated by asterisks (^***^P ≤ 0.001), ‘ns’ (no significant difference) by Student’s *t*-test. Error bars represent the standard error of the mean, and the sample size (*n*) for each group is provided. Scale bars in the microscopy images represent 50 μm.

Treatment of stigmas with 5 mM acetyl-CoA led to a significant increase in pollen attachment and pollen tube growth following compatible pollination with compatible cross *S_45_S_45_* pollen (Fig. 3A and B). Interestingly, the same treatment completely abolished the SI response in self-incompatible (*S_13-b_S_13-b_*) stigmas, as evidenced by the increased pollen attachment and pollen tube growth in a dose-dependent manner, with an average of ~230 self-pollen tubes growing into the stigmas (Fig. 3C and D).

Other than its importance in FFA synthesis, acetyl-CoA is involved in several pathways including maintaining the metabolic and energy needs for the system. To be more specific to FFA synthesis we next utilized malonyl-CoA, which is the rate-limiting and the only substrate for FA and lipid membrane synthesis. When we treated stigmas with malonyl-CoA we observed very similar outcomes to treatment with acetyl-CoA. Malonyl-CoA treatment led to breaking down of the SI response and enhanced compatible pollination, with increased pollen attachment and pollen tube growth in a dose-dependent manner (Fig. 3A–D). These findings suggest that these precursor molecules and their intermediate/final products such as FFAs might be involved in regulating SI response.

### Stigma pretreated with acetyl-CoA can contribute to the accumulation of lipids

FFAs are usually esterified into lipids, and are important constituents of membrane lipids as signaling molecules, major energy-storage reserves [20, 21]. Lipids are generally divided into eight classes: FAs, glycerolipids (GLs), glycerophospholipids (GPs), sphingolipids (SPs), sterol lipids (STs), prenol lipids (PRs), saccharolipids (SLs), and polyketides (PKs) [22, 23]. Lipid metabolism is a series of complex and highly regulated reactions for the synthesis and degradation of FAs, complex acyl lipids, and terpenoid lipids [23]. The synthesis and metabolism process between FAs and lipids is not unidirectional, but dynamically balanced.

**Figure 4:**
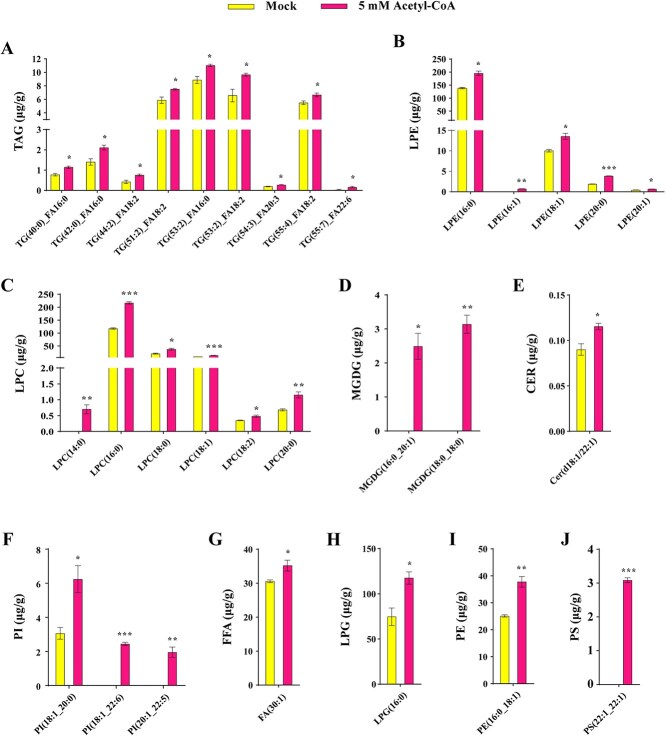
The lipid profile of stigma pretreated with 5 mM acetyl-CoA. **A.** Triacylglycerols (TAG). **B.** Lysophosphatidylethanolamines (LPE). **C.** Lysophosphatidylcholines (LPC). **D.** Monogalactosyldiacylglycerol (MGDG). **E.** Ceramides (CER). **F.** Phosphatidylinositol (PI). **G.** Free fatty acids (FFAs). **H.** Lyso-phosphatidylglycerol (LPG). **I.** Phosphatidylethanolamines (PE). **J.** Phosphatidylserine (PS).

To assess the effects of exogenous application of acetyl-CoA on lipids in stigma, we tested the lipid contents of stigma pretreated with 5 mM acetyl-CoA. As a result, stigma pretreated with 5 mM acetyl-CoA contribute to higher levels of lipid species TAG, LPE, LPC, MGDG, CER, PI, FFA, LPG, PE, and PS in stigma compared with the untreated stigma (Fig. 4A–J). A total of 30 lipid substances were found to accumulate dramatically after stigma were pretreated with 5 mM acetyl-CoA, including 9 TAG (TAG40:0_FA16:0, TAG42:0_FA16:0, TAG44:2_FA18:2, TAG51:2_FA18:2, TAG53:2_FA16:0, TAG53:2_FA18:2, TAG54:3_FA20:3, TAG55:4_FA18:2, and TAG55:7_FA22:6), 5 LPE (LPE16:0, LPE16:1, LPE18:1, LPE20:0, and LPE20:1), 6 LPC (LPC14:0, LPC16:0, LPC18:0, LPC18:1, LPC18:2, and LPC20:0), 2 MGDG (MGDG16:0_20:1 and MGDG18:0_18:0), 1 CER (d18:1/22:1), 3 PI (PI18:1_20:0, PI18:1_22:6, and PI20:1_22:5), 1 FFA (FA30:1), 1 LPG (LPG16:0), 1 PE (PE16:0_18:1), and 1 PS (PS22:1_22:1), suggesting that stigma pretreated with acetyl-CoA facilitated the formation of lipids in stigma, which can provide necessary resources for pollen germination and pollen tube growth.

### Inhibiting ACCase activity compromises the compatible pollination response

ACCase catalyzes the initial and rate-limiting step in FA synthesis, which involves the carboxylation of acetyl-CoA to malonyl-CoA. If ACCase activity in stigmas plays a role in pollination, then inhibiting this enzyme should compromise compatible pollination. To test this hypothesis, we treated stigmas with two known ACCase inhibitors, olumacostat glasaretil (OG) and acetyl-CoA carboxylase-IN-1 (ACC-IN-1), and examined their effects on pollination responses.

**Figure 5:**
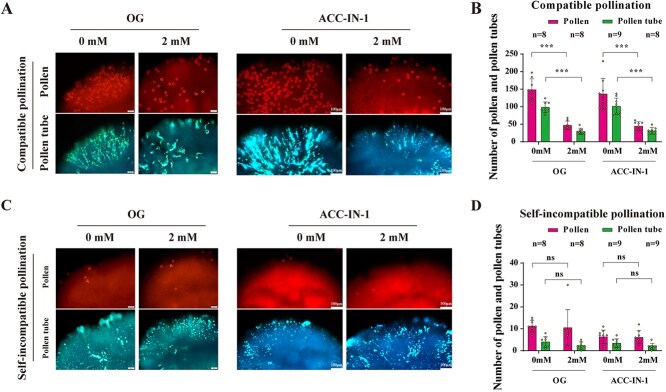
Inhibiting ACCase activity reduces compatible pollen attachment and tube growth. **A.** Fluorescence microscopy images showing pollen and pollen tube growth on *S_13-b_S_13-b_* stigmas following CP. The stigmas were treated with either 0 mM (control) or 2 mM of OG or ACC-IN-1. **B.** Quantification of pollen attachment and pollen tube growth following CP. The data shows a significant reduction in both pollen attachment and pollen tube growth with 2 mM OG or ACC-IN-1. **C.** Fluorescence microscopy images showing pollen and pollen tube growth on *S_13-b_S_13-b_* stigmas following self-incompatible pollination. The stigmas were treated with either 0 mM (control), 2 mM of OG or ACC-IN-1. **D.** Quantification of pollen attachment and pollen tube growth with OG or ACC-IN-1 treatment following self-incompatible pollination. The data shows no significant changes in pollen attachment or pollen tube growth. Statistical significance is indicated by asterisks (^***^P ≤ 0.001), ‘ns’ (no significant difference) by Student’s *t*-test. Error bars represent the standard error of the mean, and the sample size (*n*) for each group is provided. Scale bars in the microscopy images represent 50 μm (left) or 100 μm (right).

We pretreated stigmas with ACCase inhibitors (OG or ACC-IN-1), and then detected ACCase activity in the stigma. As expected, 2 mM OG or 2 mM ACC-IN-1 treatment can effectively suppress ACCase activity in stigma (Supplementary Fig. S4). Treatment of stigmas with 2 mM OG or 2 mM ACC-IN-1 resulted in a significant reduction in pollen attachment and germination following compatible pollination (Fig. 5A and B). These treatments did not have a significant effect on pollen attachment following self-incompatible pollination (Fig. 5C and D). These findings suggest that stigmatic ACCase activity is required for compatible pollination responses.

### BCCP1, a functional subunit of ACCase, interacts with FERONIA

ACCase is a crucial enzyme in the FA synthesis pathway. Our results demonstrate that treatment of stigmas with acetyl-CoA or malonyl-CoA can completely break down the SI response, while inhibiting ACCase can reduce compatible pollination. ACCase is a multi-subunit enzyme composed of four components: biotin carboxyl-carrier protein (BCCP), biotin carboxylase, α-carboxyltransferase, and β-carboxyltransferase [24]. In *Arabidopsis*, AtBCCP1 and AtBCCP2 are the primary key components of ACCase, with AtBCCP1 playing a regulatory role in embryo, endosperm, and pollen development [24].

ACCase is encoded by BCCP1 and BCCP2 in ornamental kale, sequence analysis revealed limited homology between BoBCCP1 and BoBCCP2, with only 44.68% amino acid sequence similarity (Supplementary Fig. S5A). Our full-length transcriptome analysis of stigma development stages (small: 0–4 mm; medium: 6–8 mm; large: ≥10 mm) in the self-incompatible line *S_13-b_S_13-b_* [25] revealed distinct expression patterns for BoBCCP1 and BoBCCP2 (Supplementary Fig. S5B). BoBCCP1 expression was the highest in immature stigmas and decreased progressively during development, while BoBCCP2 showed a peak expression in medium-stage stigmas.

**Figure 6:**
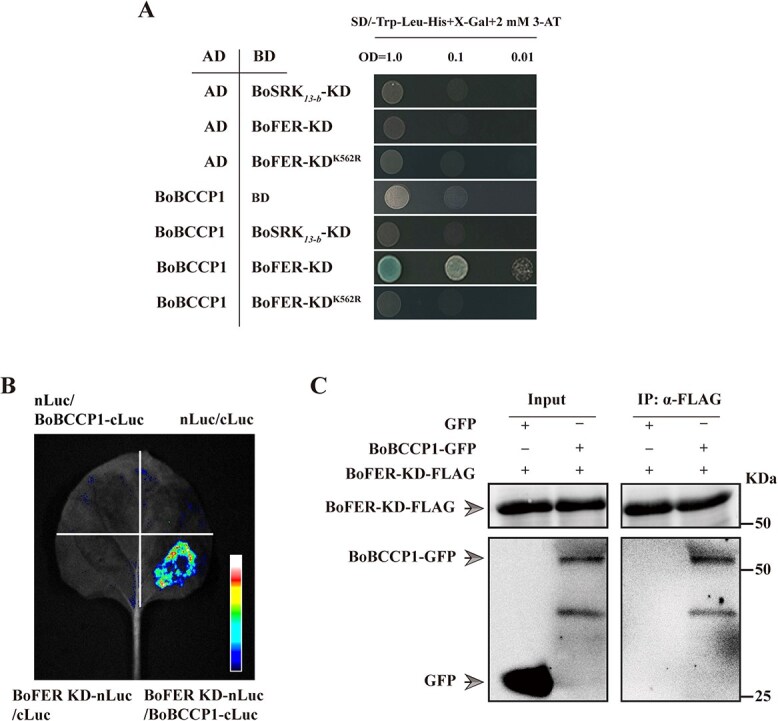
BoBCCP1, a functional subunit of ACCase, interacts with BoFER. **A.** Y2H assay showing BoBCCP1 interaction with BoFER-KD. The results indicate that BoBCCP1 interacts with BoFER, as evident from the yeast growth on the selective medium (SD/−Trp-Leu-His+X-gal+2 mM 3AT). **B.** Split luciferase complementation assay in *N. benthamiana* leaves showing BoBCCP1 interaction with BoFER-KD. Agrobacterium expressing nLuc, cLuc, BoBCCP1-cLuc, and BoFER-KD-nLuc as indicated were infiltrated into *N. benthamiana* leaves. After 72 h, luciferin was injected onto the inoculated leaves. Luminescence signals were detected 10 min post-infiltration with chemiluminescent imaging (Tanon) apparatus. The luminescence image shows a strong signal for the BoBCCP1-cLuc and BoFER-KD-nLuc combination, confirming their interaction in plant cells. Other combinations served as controls. **C.** CoIP assay showing BoBCCP1 interaction with BoFER-KD. Co-expression of BoFER-KD-3xFLAG and BoBCCP1-GFP in tobacco leaves. Total proteins were extracted and immunoprecipitated with anti-FLAG antibody, then subjected to WB assay with anti-GFP antibodies. IP: immunoprecipitation.

Given the recent discoveries of new receptor modules, such as the SRK-FER complex, we investigated whether any of the ACCase subunits interact with FER or SRK. Yeast two-hybrid assays (Y2H) revealed that BoBCCP1 interacted with the kinase domain of BoFER (BoFER-KD) but not with BoSRK (BoSRK-KD) (Fig. 6A). Furthermore, luciferase complementation assays (LUC) and co-immunoprecipitation (Co-IP) assays confirmed the direct interaction between BoFER-KD and BoBCCP1 (Fig. 6B and C). BoBCCP2 did not show the interaction with BoFER-KD in Y2H and Co-IP assays (Supplementary Fig. S6A and B). Consider: K565 of the *Arabidopsis* AtFER is an essential catalytic site for its kinase activity. [26]. We observed that a point mutant in the analogous location of BoFER eliminated the interaction (Fig. 6A), suggesting that the interaction between BoFER and BoBCCP1 is dependent on BoFER kinase activity.

These findings raise the possibility that the interaction between BoBCCP1 and BoFER may play a negative role in regulating FA synthesis during pollination. The dependence of this interaction on FER kinase activity suggests a potential mechanism for the regulation of ACCase activity and, consequently, the production of downstream FAs essential for successful pollination.

### BCCP1 involved in compatible pollination

**Figure 7:**
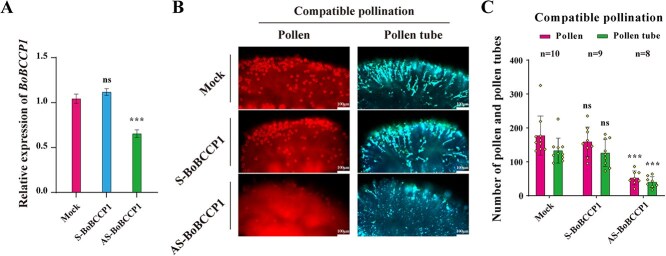
BoBCCP1 participated in CP. **A.** RT-qPCR analysis of *BoBCCP1* expression in *S_13-b_S_13-b_* stigmas treated with sense (S-) or antisense (AS-) *BoBCCP1* RNA. The data shows significant downregulation of BoBCCP1 in stigmas treated with AS-BoBCCP1 compared to the mock and S-BoBCCP1 treatments. **B.** Fluorescence microscopy images showing pollen and pollen tube growth on *S_13-b_S_13-b_* stigmas following CP. The images compare mock treatment with S-BoBCCP1 or AS-BoBCCP1 treatments. Reduced pollen attachment and pollen tube growth are observed in the AS-BoBCCP1 treatment. **C.** Quantification of pollen attachment and pollen tube growth following CP with S-BoBCCP1 or AS-BoBCCP1 treatments. The data shows a significant reduction in both pollen attachment and pollen tube growth with AS-BoBCCP1 treatment. Statistical significance is indicated by asterisks (^***^P ≤ 0.001), ‘ns’ (no significant difference) by Student’s *t*-test. Error bars represent the standard error of the mean, and the sample size (*n*) for each group is provided. Scale bars in the microscopy images represent 100 μm.

To functionally analyze the role of *BoBCCP1* in pollination, we treated self-incompatible stigmas with an antisense oligodeoxyribonucleotide (AS-ODN) specifically targeting *BoBCCP1* (Supplementary Fig. S7A and B). Quantitative real-time polymerase chain reaction (RT-qPCR) analysis confirmed the suppressed expression of *BoBCCP1* in treated stigmas (Fig. 7A). Following compatible pollination, we observed a significant reduction in the number of pollen grains and pollen tube growth in stigmas treated with AS-BoBCCP1 (Fig. 7B and C), implying that *BoBCCP1* is key for successful pollen–stigma interactions. Reduction in BoBCCP1, as expected, did not affect the SI response (Supplementary Fig. S8A and B). These results support the hypothesis that the BoFER-BoBCCP1 signaling module may mediate optimal ACCase activity necessary for compatible pollen growth. As demonstrated earlier, BoBCCP1 interacts with the kinase domain of BoFER (Fig. 6A–C), suggesting a potential mechanism for the regulation of ACCase activity during pollination.

## Discussion

Our study reveals that compatible pollination predominantly drives alterations in α-linolenic acid and linoleic acid metabolism, reflecting a more pronounced induction of FFA metabolism during successful pollination. This finding underscores the importance of lipids in the pollination process, which has been previously demonstrated in various plant species. For example, in Solanaceae, lipids have been shown to be essential for directional pollen tube growth and penetration of the stigma [27]. Similarly, in *Brassica napus*, lipidic components in both sporophytic and gametophytic tissues contribute to the production of viable and fertile pollen grains [28]. Furthermore, lipid-rich secretions on the stigma surface have been proposed to establish a water potential gradient at the pollen–stigma interface, facilitating water uptake by the pollen and guiding pollen tube growth toward the ovule [27, 29, 30].

Previous studies have primarily focused on the role of lipids in various aspects of pollination, such as pollen development [31, 32], male sterility [33, 34], and stigma receptivity [27]. However, the dynamic changes in FFAs during the early postpollination events and their significance in regulating both CP and SI responses have not been explored until now. Our study is the first to report the specific alterations in FFA levels during the initial stages of pollination and to demonstrate their crucial role in determining the success of pollination. By employing an integrated analysis of metabolite levels, we not only corroborate and extend the previous findings on the importance of lipids in pollination but also provide novel insights into the molecular mechanisms underlying the regulation of pollen–stigma interactions.

Acetyl-CoA, generated through glycolysis and subsequently entering the tricarboxylic acid (TCA) cycle, plays a crucial role in providing energy for pollen germination and tube growth [35, 36]. Interestingly, our study found that exogenous treatment with acetyl-CoA or malonyl-CoA not only promoted CP but also broke down SI. This finding suggests that these metabolites may have a direct impact on the pollen–stigma recognition process beyond their role in energy production. Surprisingly, after treatment with 5 mM acetyl-CoA, the number of self-pollen tubes was higher than that of cross-pollen tubes in the stigmas. Further research is needed to clarify the extent to which acetyl-CoA treatment can disrupt pollen–stigma recognition across diverse plant taxa and to elucidate the underlying molecular mechanisms.

ACCase catalyzes the ATP-dependent carboxylation of acetyl-CoA to generate malonyl-CoA, which is a rate-limiting step in *de novo* FA synthesis. Our inhibitor assays with ACCase demonstrate that ACCase activity in stigmas is required for compatible pollination, as the inhibition of ACCase led to a significant reduction in pollen attachment and germination. These findings are consistent with the observed downregulation of FA biosynthesis pathways in mature self-incompatible stigmas [25] and their upregulation during CP. The differential regulation of these pathways suggests that the levels of FAs play a crucial role in determining the success of pollination. The downregulation of FA biosynthesis in self-incompatible stigmas may contribute to the rejection of self-pollen, while the upregulation of these pathways during compatible pollination may facilitate the acceptance and growth of compatible pollen.

Recent studies have shown that the stigma FER-SRK complex plays a crucial role in regulating SI responses in Brassicaceae species, such as Chinese cabbage, through the modulation of ROS production [6, 7]. In addition to its function in SI, FER has been proposed to act as a scaffold in receptor complexes and mediate compatible pollination responses following intergeneric hybridization with specific RALF peptides in *Arabidopsis* [8]. Our results reveal that BoBCCP1, a homolog of AtBCCP1 in *Arabidopsis*, interacts with the kinase domain of BoFER. In *Arabidopsis*, AtBCCP1 and AtBCCP2 exhibit unidirectional functional redundancy; the absence of AtBCCP1 cannot be tolerated as AtBCCP2 levels are not sufficient to support ACCase activity; AtBCCP1 can compensate for the loss of AtBCCP2, but AtBCCP2 cannot replace AtBCCP1; this asymmetric relationship is reflected in the finding that reduction of AtBCCP1 levels, but not AtBCCP2, significantly affects FAs accumulation; it was reported that AtBCCP1 is an essential subunit for ACCase; and AtBCCP1 can affect embryo and endosperm development, pollen grains germination, and pollen tube growth [24].

Sequence analysis revealed that BCCP1 and BCCP2 share low amino acid sequence homology (44.68% similarity) (Fig. S5A). This divergence suggests potential functional specialization, similar to what has been observed in *Arabidopsis*. Our full-length transcriptome analysis of stigma development stages [25] also revealed that both BoBCCP1 and BoBCCP2 have different expression patterns in stigmatic tissues (Fig. S5B). BoBCCP1 expression was the highest in immature stigmas and decreased progressively during development, while BoBCCP2 showed a peak expression in medium-stage stigmas. These differential expression patterns suggest stage-specific functions during stigma development and maturation. The sequence and expression pattern differences of BCCP1/2 may lead to differential interactions with BoFER. The differential interaction with BoFER adds another layer of regulation, potentially allowing for fine-tuning of ACCase activity in response to pollination signals. This regulatory mechanism appears to target BoBCCP1, highlighting its predominant role in controlling ACCase activity during pollination.

**Figure 8:**
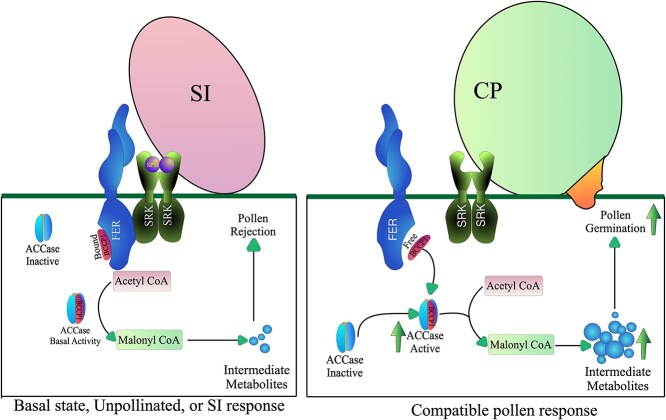
A proposed model of the FER-BCCP module regulating SI and CP response. The conversion of acetyl-CoA to malonyl-CoA by ACCase is a critical rate-limiting step. BCCP1, the main ACCase subunit, interacts with FER, which renders ACCase inactive, resulting in impaired intermediate metabolites synthesis, which in turn leads to self-pollen rejection. On the contrary, during compatible response, free BCCP1 binds to ACCase restoring an increased activity to convert acetyl-CoA to malonyl-CoA and recharging the intermediate metabolites, thereby providing the necessary resources for pollen germination and pollen tube growth.

Interestingly, we found that BoBCCP1 does not interact with a kinase-dead mutant of BoFER (BoFER-KD^K562R^), suggesting that the interaction between BoFER and BoBCCP1 depends on BoFER kinase activity. Given that FER is required for SI response and the default stigmatic state may be an SI-primed state, there is basal activation of FER in the system that could be hyperactivated following SCR/SRK activation. FER activity likely leads to sequestration of BCCP1 and the inactivation of ACCase, resulting in reduced basal FFA synthesis and self-pollen rejection. Conversely, following compatible pollination, FER kinase activity is compromised by binding of PCPs, which allows BCCP1 to interact with other ACCase components, restoring increased ACCase activity and FFA production, thus providing the necessary resources for pollen germination and pollen tube growth (Fig. 8).

Our research suggests a downstream signaling event triggered by the FER-BCCP1 interaction for accepting compatible pollen or rejecting incompatible pollen. Understanding these pathways could reveal additional targets for manipulating SI responses, thus expanding the possibility of facilitating hybrid breeding and improving agricultural yields. For example, by modulating the expression or activity of key components in the FER-BCCP1 signaling pathway, it may be possible to overcome SI barriers and promote hybridization between otherwise incompatible plant varieties. Additionally, the identification of metabolites or precursors that can break down SI, such as acetyl-CoA and malonyl-CoA, could be exploited to develop novel strategies for controlling pollination in agriculturally important crops. However, further research is needed to investigate the role of these precursors and metabolites in overcoming inter- or intraspecific reproductive barriers and to assess their potential applications in agricultural practices.

In conclusion, our study provides compelling evidence for the crucial role of FA metabolism in regulating pollination success and sheds light on the molecular mechanisms underlying the FER-BCCP1 signaling pathway in mediating SI and CP responses. The integration of metabolomic and biochemical approaches has allowed us to unravel the complex interplay between lipid signaling and pollen–stigma interactions. While our findings represent a significant advancement in our understanding of pollination biology, they also open up new avenues for future research. Investigating the conservation of the FER-BCCP1 signaling pathway across diverse plant species, elucidating the downstream targets of this pathway, and exploring the potential applications of metabolite-based approaches for manipulating pollination are just a few examples of the exciting research directions that could stem from this study. Ultimately, our work lays the foundation for future investigations into the intricate molecular dialogs that govern plant reproduction and highlights the importance of integrating metabolomic approaches in deciphering the complex mechanisms underlying pollination success.

## Materials and methods

### Plant materials

Self-incompatible line *S_13-b_S_13-b_* and compatible line *S_45_S_45_* of ornamental kale (*Brassica oleracea* var. *acephala*) were selected for this study due to their well-characterized SI and compatibility responses [10]. Plants were vernalized during the seedling stage and then grown in a greenhouse at the Flower Bioengineering Institute of Northeast Forestry University. For incompatible and compatible pollinations, mature stigmas at the anthesis stage from *S_13-b_S_13-b_* plants were collected after emasculation and hand-pollinated with either *S_13-b_S_13-b_* pollen (SI pollination) or *S_45_S_45_* pollen (CP). Pistils were collected at different time points (0, 30, and 60 min) following pollination, immediately flash-frozen in liquid nitrogen, and stored at −80°C for metabolome analysis. Pistils from each time point and pollination treatment were pooled to create three biological replicates for metabolomic analysis.

### Widely targeted metabolomics analysis

Metabolite extraction and analysis were performed by Wuhan Metware Biotechnology Co., Ltd (Wuhan, China) following standard protocols [37, 38]. Briefly, lyophilized ornamental kale pistil samples (50 mg) were ground using a mixer mill (MM 400, Retsch) with a zirconia bead for 1.5 min at 30 Hz and extracted with 1.2 ml 70% methanol. The extracted samples were analyzed using a UPLC-ESI-MS/MS system (UPLC: SHIMADZU Nexera X2, https://www.shimadzu.com.cn/; MS: Applied Biosystems 4500 QTRAP, http://www.appliedbiosystems.com.cn/). Metabolite identification and quantification were performed based on a self-built metware database (MWDB) using scheduled multiple reaction monitoring (MRM). Orthogonal projections to latent structures discriminant analysis (OPLS-DA) was conducted using the OPLSR.Anal function in the MetaboAnalystR package to screen for DMs. DMs were selected based on VIP ≥ 1 and |Log2FC (fold change)| ≥1. The identified DMs and metabolic pathways were annotated using the KEGG compound database (http://www.kegg.jp/kegg/compound/) and KEGG pathway database (http://www.kegg.jp/kegg/pathway.html).

### Excised stigma-feeding assays

Mature stigmas from self-incompatible *S_13-b_S_13-b_* plants were used for the study. Following emasculation, stigmas were cut 2–3 mm below the stigmatic surface and inserted into a modified PGM medium [6] containing different concentrations of acetyl-CoA, malonyl-CoA, olumacostat glasaretil [39,], or acetyl-CoA carboxylase-IN-1 [40]. Control medium contained an equal amount of the relevant solvent. After a 3-h treatment, stigmas were pollinated with either *S_13-b_S_13-b_* pollen (self-incompatibe pollination) or *S_45_S_45_* pollen (CP). Six hours postpollination, stigmas were subjected to aniline blue staining, and pollen grain attachment and pollen tube growth were visualized using an OLYMPUS BX43F microscope (Tokyo, Japan).

### Protein interaction assays

For Y2H assays, full-length cDNAs of *BoBCCP1* and *BoBCCP2* were cloned into the pGADT7 (AD) vector, while cDNAs encoding the kinase domains of *BoFER* (BoFER-KD) and *BoSRK_13-b_* (*BoSRK_13-b_*-KD) were cloned into the pGBKT7 (BD) vector. The recombinant AD and BD plasmid pairs were co-transformed into the yeast strain Y2HGold and plated on SD/−Trp-Leu medium. Positive transformants were spotted in 10-fold serial dilutions on SD-Leu-Trp and SD-Leu-Trp-His media and cultured at 30°C for 3–4 days. The BoFER-KD (K562R) mutant was generated using the KOD-PlusMutagenesis Kit (TOYOBO).

For luciferase complementation imaging assays, cDNAs encoding BoFER-KD and BoSRK*_13-b_*-KD were cloned into the pCAMBIA 1300-nLuc vector, while full-length *BoBCCP1* cDNA was cloned into the pCAMBIA 1300-cLuc vector. The recombinant plasmid pairs were transformed into *Agrobacterium tumefaciens* GV3101, and the transformed strains were mixed at equal densities (OD_600_ = 1.0) and injected into *Nicotiana benthamiana* leaves. After 72 h, 1 mM luciferin was injected into the inoculated leaves, and luminescence signals were captured using a Tanon chemiluminescent imaging system.

For Co-IP and western blot (WB) assays, *BoBCCP1* and *BoBCCP2* were cloned into the pGD3GGm vector to generate BoBCCP1-GFP and BoBCCP2-GFP constructs, while BoFER-KD was cloned into the pGD3G3Flag vector to generate the BoFER-KD-3xFLAG construct. The recombinant plasmids were transformed into *A. tumefaciens* GV3101, and each interaction pair was mixed with the P19 silencing suppressor at a ratio of 5:5:3 before injection into *N. benthamiana* leaves. After 48 h, leaf samples were collected, and total proteins were extracted and immunoprecipitated using anti-FLAG or anti-GFP magnetic beads (Sigma Aldrich). The immunoprecipitated proteins were analyzed by WB using anti-GFP and anti-FLAG antibodies (Abmart), and the signals were detected using a Tanon 4600 system.

For all expression vectors, the presence of the desired inserts was confirmed by PCR, and sequence accuracy was verified by Sanger sequencing. The primers used for molecular cloning are listed in Supplementary Table S7.

### ODN design and treatment

Sense and antisense oligodeoxyribonucleotides (S-ODN and AS-ODN) targeting *BoBCCP1* were designed as previously described [6, 41]. The 5′ and 3′ ends of the ODNs were modified with three phosphorothioate bonds to enhance stability. The ODN sequences are listed in Supplementary Table S7. ODN treatment of stigmas was performed as described for the stigma feeding assays [6, 37] with minor modifications. Stigmas from newly opened flowers were excised 2–3 mm below the stigmatic surface and inserted into PGM containing either S-ODN or AS-ODN. After a 2.5-h treatment, stigmas were pollinated with either *S_13-b_S_13-b_* pollen (SI pollination) or *S_45_S_45_* pollen (CP). Six hours postpollination, stigmas were subjected to aniline blue staining, and pollen grain attachment and pollen tube growth were observed using a LEICA DM4 B fluorescence microscope.

### RT-qPCR analysis

Total RNA was extracted using the Plant RNA Kit (OMEGA), and cDNA was synthesized using the TransScript One-Step gDNA Removal and cDNA Synthesis SuperMix (TransGen). RT-qPCR was performed using TransStart Top Green qPCR SuperMix (TransGen) on a LightCycler480 System (Roche). The relative expression of target genes was normalized to the internal control *ACTIN*. Three biological replicates were performed for each RT-qPCR experiment, and the 2^-ΔΔCT^ method was used to analyze the expression levels of the target genes. RT-qPCR primers were designed using Primer Premier 5.0, and the primer sequences are listed in Supplementary Table S7.

### Acetyl-CoA carboxylase activity assay

ACCase activities were measured and analyzed using the commercial enzyme activity assay kits of Suzhou Mengxi Biomedical Technology Co., Ltd. (https://www.michybio.com) according to manufacturer’s instructions.

### Lipid extraction and quantification analysis

Lipid extraction and UPLC–MS/MS quantification analysis were carried out on stigma pretreated with acetyl-CoA by Biotree Biomedical Technology Co., Ltd. (https://www.biotree.cn/, Shanghai, China). In brief, the sample is freeze-dried and ground into a powder, 10 mg of the sample was weighted, and after the addition of 400 μl water was vortexed for 60 s, homogenized at 45 Hz for 4 min, and sonicated for 5 min. Twenty microliters of homogenate was mixed with 180 μl of water, and then 480 μl of extraction solution containing internal standard (MTBE:MeOH = 5:1) was added. The sample was vortexed for 60 s and sonicated for 5 min. After centrifugation at 3000 rpm for 15 min at 4°C, 500 μl of supernatant was dried under vacuum; 150 μl of resuspension buffer (DCM: MeOH: H2O = 60:30:4.5) was added, the sample was vortexed for 30 s and sonicated for 10 min, and then centrifuged at 12 000 rpm for 15 min at 4°C. Seventy microliters of the supernatant was transferred to the injection vial for LC–MS analysis to detect lipid metabolite.

The chromatographic separation of the target compounds was carried out using a Nexera LC-40 series UHPLC System. The mobile phase A consisted of 40% water, 60% acetonitrile, 10 mmol/l ammonium acetate, and 0.1% acetic acid. While phase B consisted of 10% acetonitrile and 90% isopropanol, 10 mmol/l ammonium acetate, and 0.1% acetic acid. The column temperature was 45°C, and the injection volume was 2 μl. MS was performed in MRM mode, typical ion source parameters were: IonSpray Voltage: +5500/−4500 V, Curtain Gas: 50 psi, Temperature: 450°C, Ion Source Gas 1: 35 psi, Ion Source Gas 2: 50 psi. The raw mass spectrum data were converted to mzXML format using ProteoWizard software. XCMS was used for retention time correction, peak identification, extraction, integration, and alignment. Lipid identification was carried out based on LipidBlast library within the XCMS software. SCIEX Analyst Work Station Software (Version 1.6.3) and DATA DRIVEN FLOW (Version 2.0.3.11) were employed for the quantification of the target compounds. Screening and analysis of lipid metabolites were based on VIP >1 and an adjusted *P*-value (*P*adj) <0.05. Three biological replicates were performed for each experimental group.

### Quantification and statistical analysis

The experimental data were statistically analyzed using three or more averages. Statistical analysis was done using GraphPad Prism 8.0. The significance was assessed by two-tailed Student’s *t*-tests. ^*^P ≤ 0.05, ^**^P ≤ 0.01, ^***^P ≤ 0.001.

## Supplementary Material

Web_Material_uhaf147

## Data Availability

The main data supporting the findings of this study are available in the main text and supporting information of this article.
