# Supplementary material for: Free fatty acid biosynthesis precursors are involved in pollen–stigma interactions in Brassica
Source: Hortic Res. 2025 Jun 11;12(9):uhaf147. doi: 10.1093/hr/uhaf147 (PMC12313337; doi:10.1093/hr/uhaf147)
Supplement: Web_Material_uhaf147 [file web_material_uhaf147.zip › HR-Supplementary Data Figures.docx]

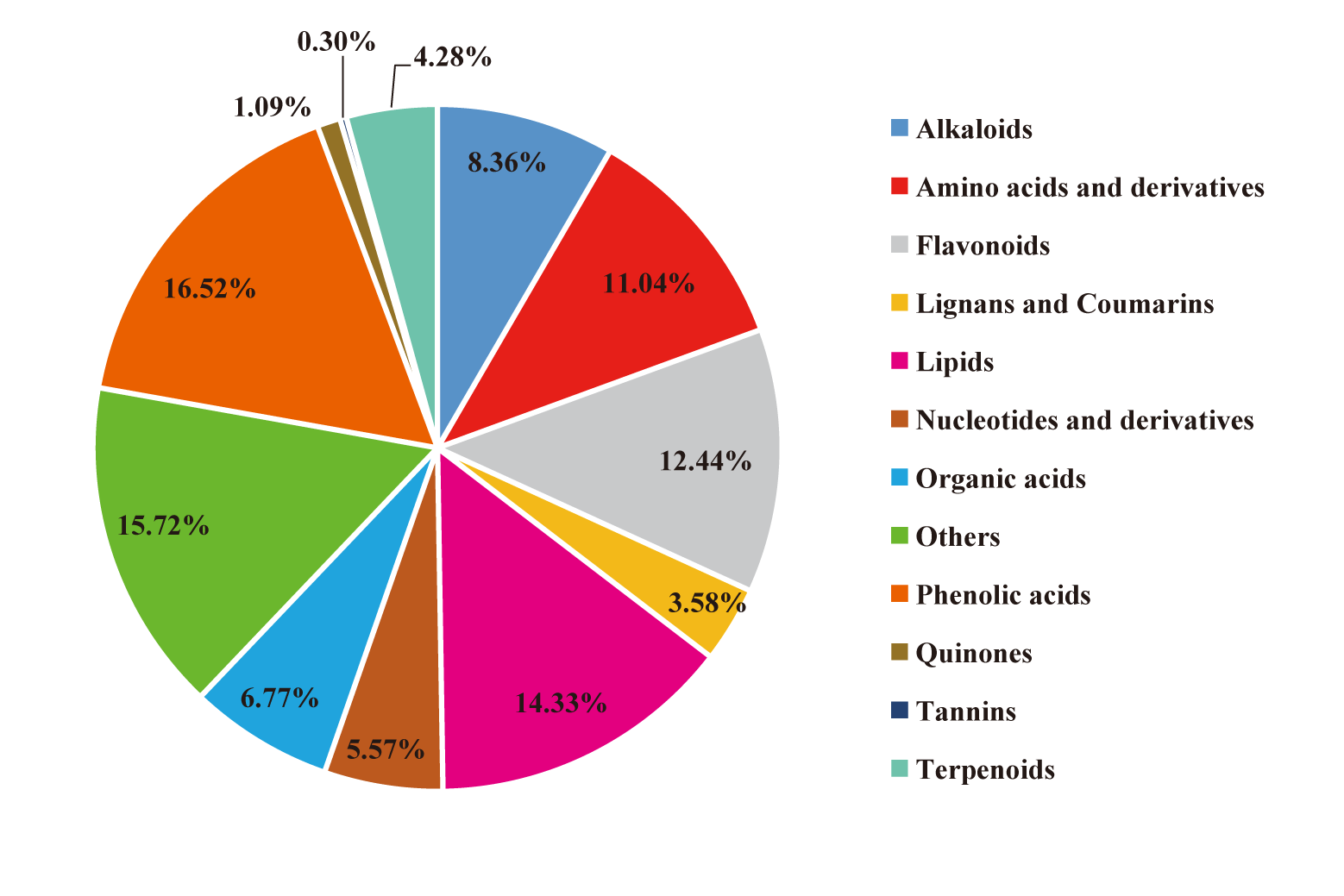


**Supplementary Data Figure S1.** Pie chart illustrating the composition of metabolite categories following pollination.


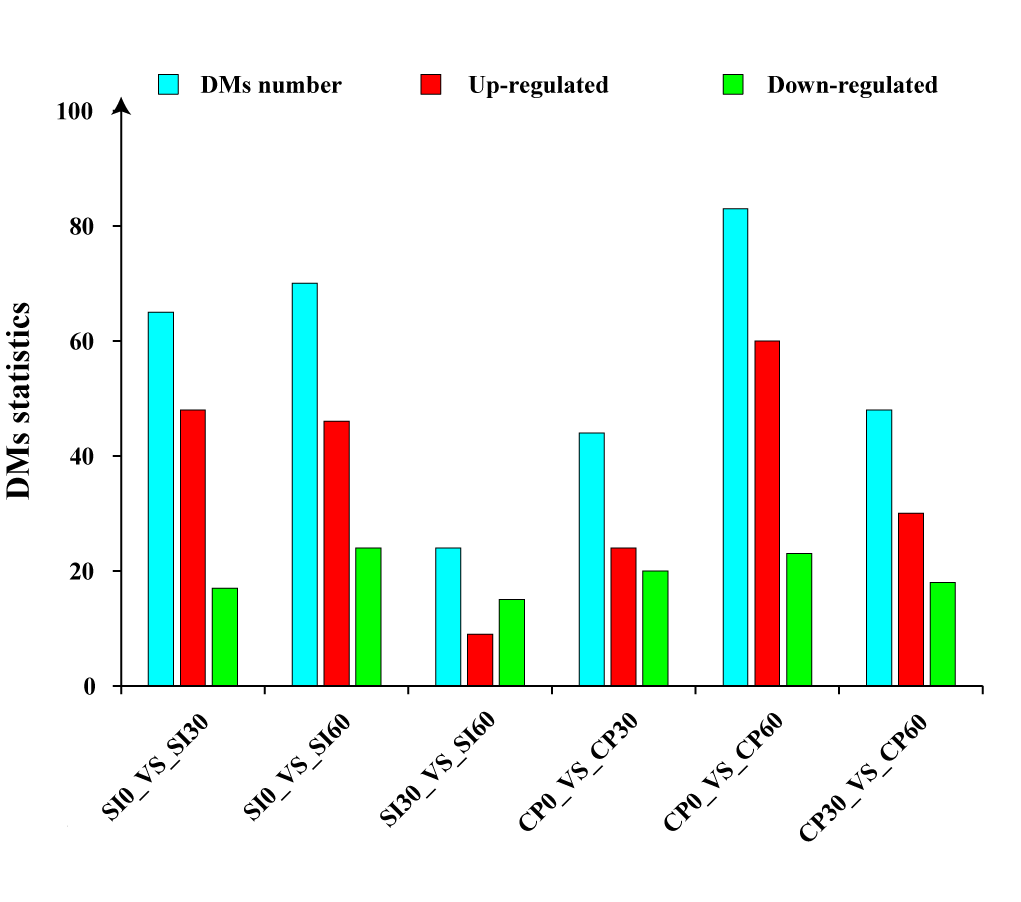


**Supplementary Data Figure S2.** Bar graph showing the number of DMs in various comparison groups at different time points (0 min, 30 min, 60 min) after pollination. The graph displays the total number of DMs, as well as the number of up-regulated and down-regulated metabolites for each comparison.


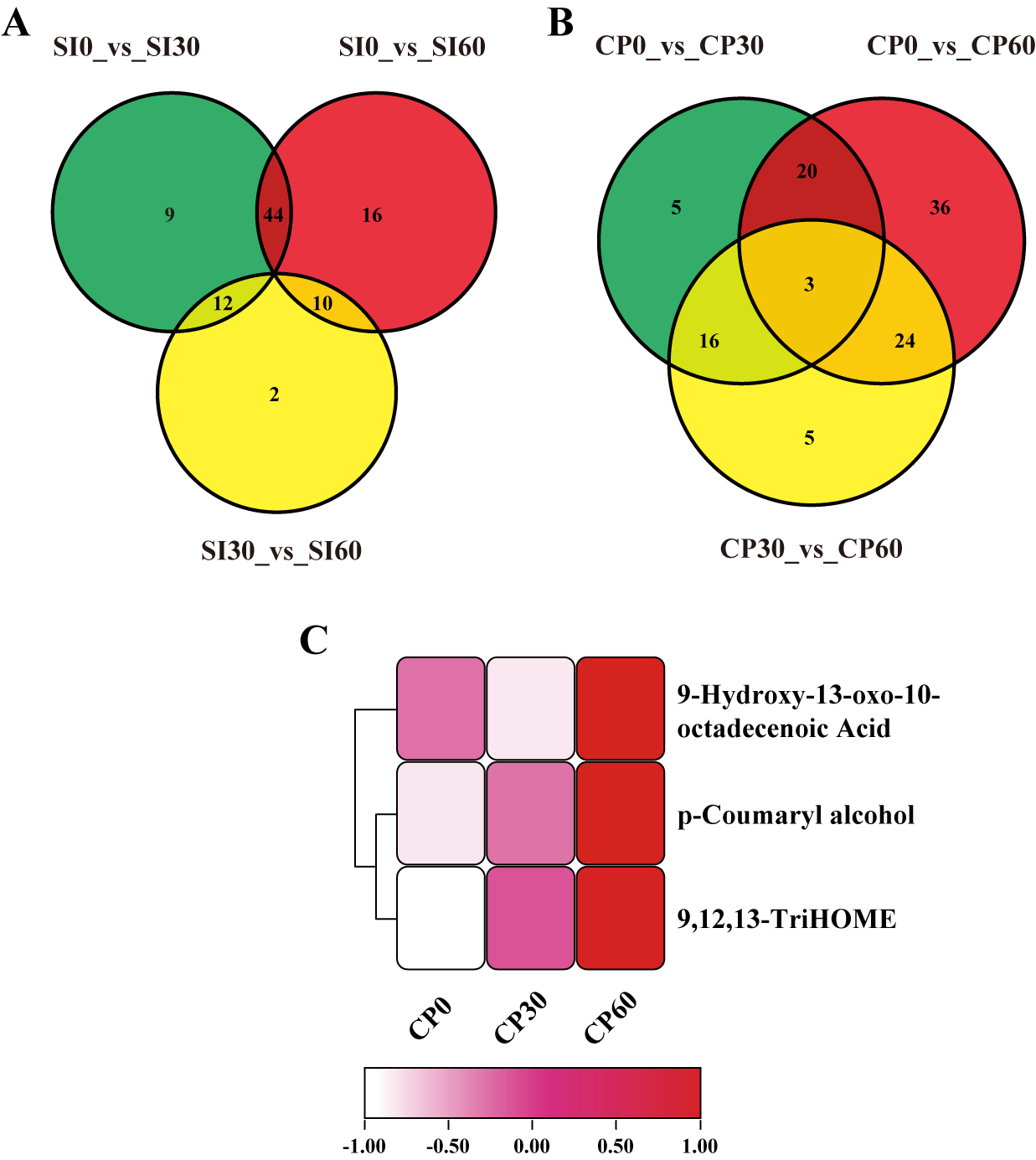


**Supplementary Data Figure S3.** Venn diagram analysis of DMs following pollination. **A** and (**B)** Venn diagram depicting the overlap of DMs among three comparison groups following self-incompatible (SI) and compatible pollination (CP), respectively. **C** Heat map displaying the expression patterns of 3 common DMs identified across different comparison groups following CP.


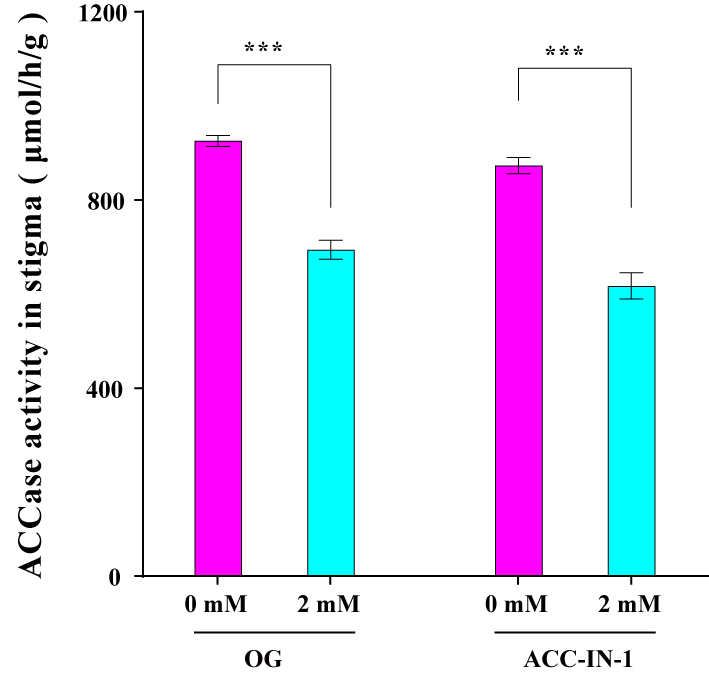


**Fig. S4.** ACCase activity of self-incompatible *S_13-b_S_13-b_* stigmas were treated with 2 mM OG or 2 mM ACC-IN-1. Statistical significance is indicated by asterisks (*** p ≤ 0.001) by Student’s t-test. Error bars represent the standard error of the mean.


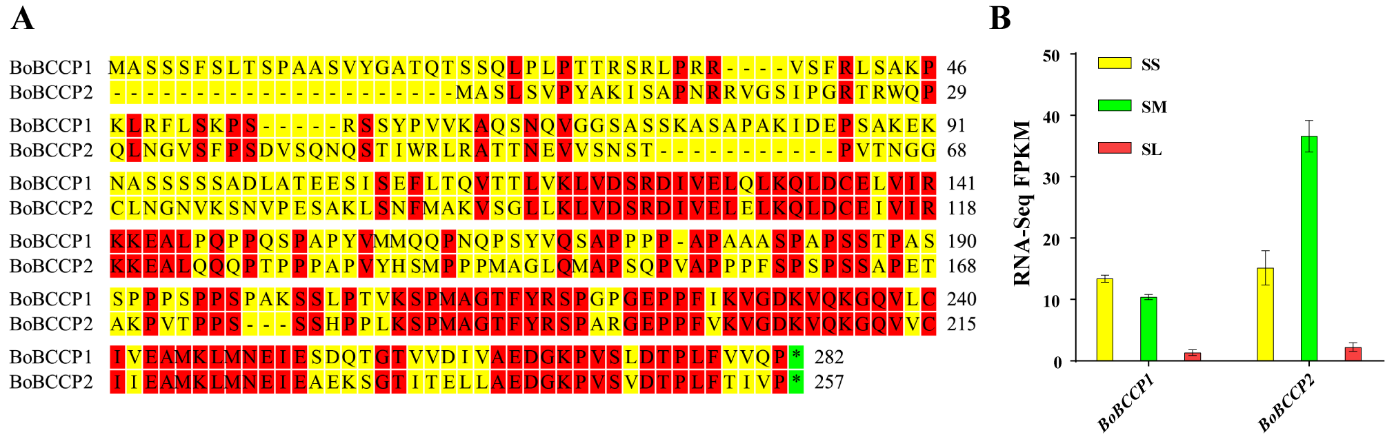


**Figure S5.** Analysis of amino acid sequence and transcriptional levels of BoBCCP1/2. **A** Amino acid sequence of BoBCCP1/2. **B** Transcriptional levels of *BoBCCP1/2* in different stages of stigma development. Error bars represent the standard error of the mean.


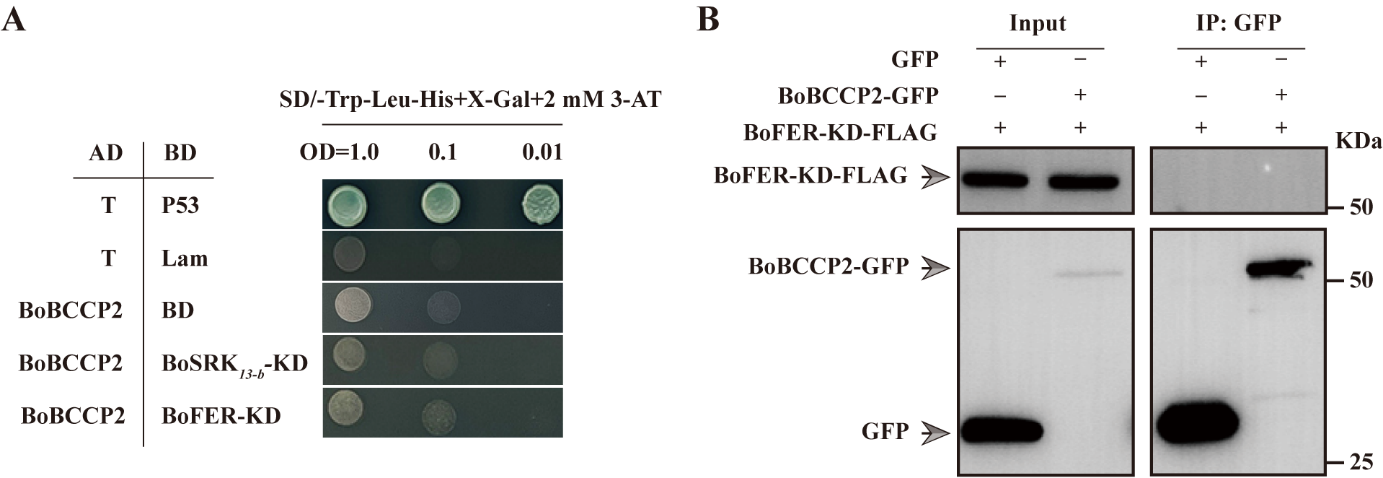


**Supplementary Data Figure S6.** BoBCCP2 didn’t show the interaction with FER in Y2H and Co-IP assays. **A** Yeast two-hybrid assay results showing no interaction between BoBCCP2 and BoFER-KD, BoSRK_13-b_-KD. The panel shows growth on SD/-Trp-Leu-His+X-gal+2 mM 3-AT medium (selective). **B** Co-immunoprecipitation (CoIP) assays showing no interaction between BoBCCP2 and FER-KD. Co‐expression of BoFER-KD-3xFLAG and BoBCCP2-GFP in tobacco leaves. Total proteins were extracted and immunoprecipitated with anti‐GFP antibody, then subjected to western blot assay with anti‐FLAG antibodies. IP: immunoprecipitation.


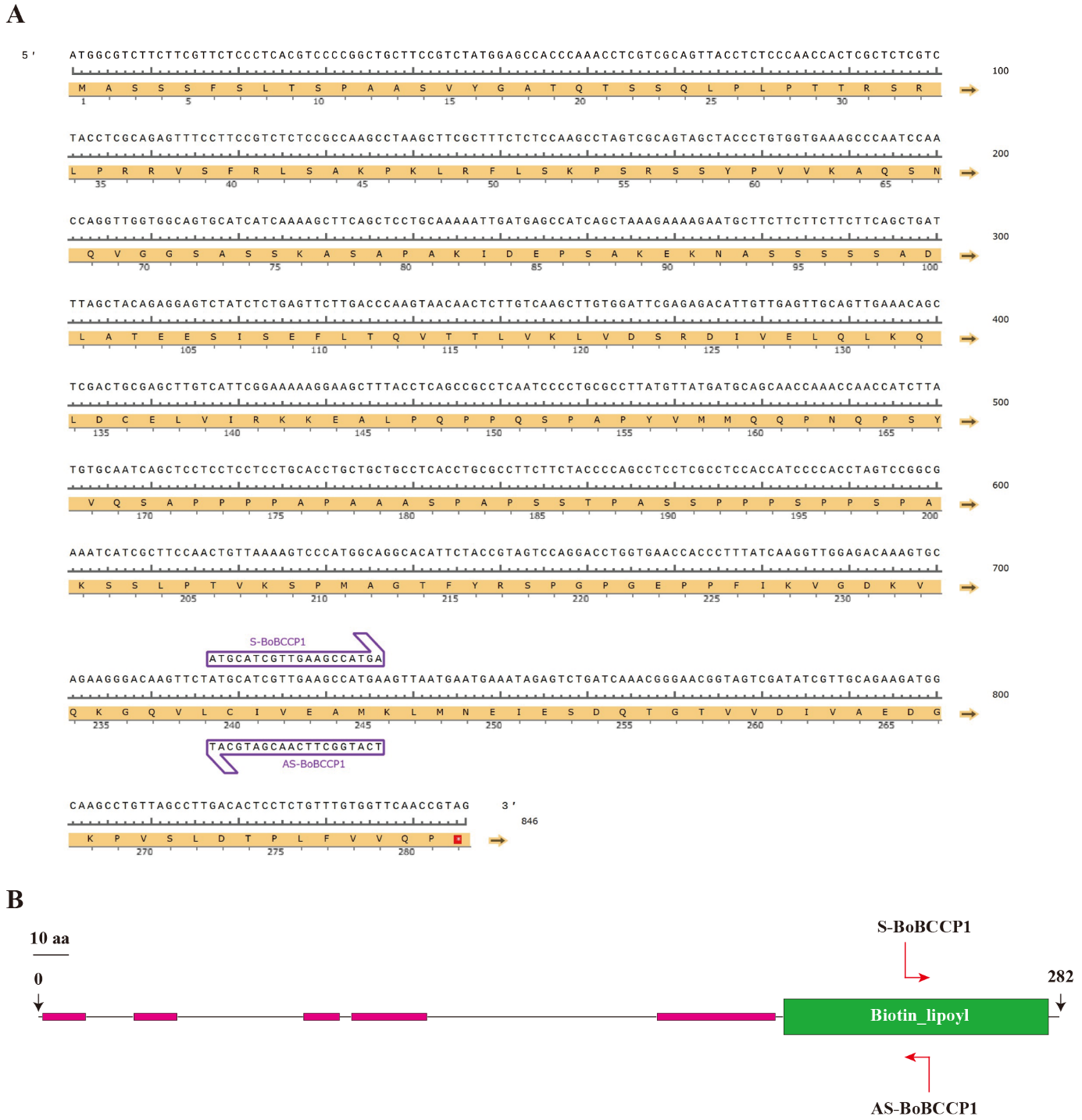


**Supplementary Data Figure S7.** S-BoBCCP1 and AS-BoBCCP1 sequences and location map. **A** S-BoBCCP1 and AS-BoBCCP1 sequences location map. **B** Domain structure of BoBCCP1 proteins.


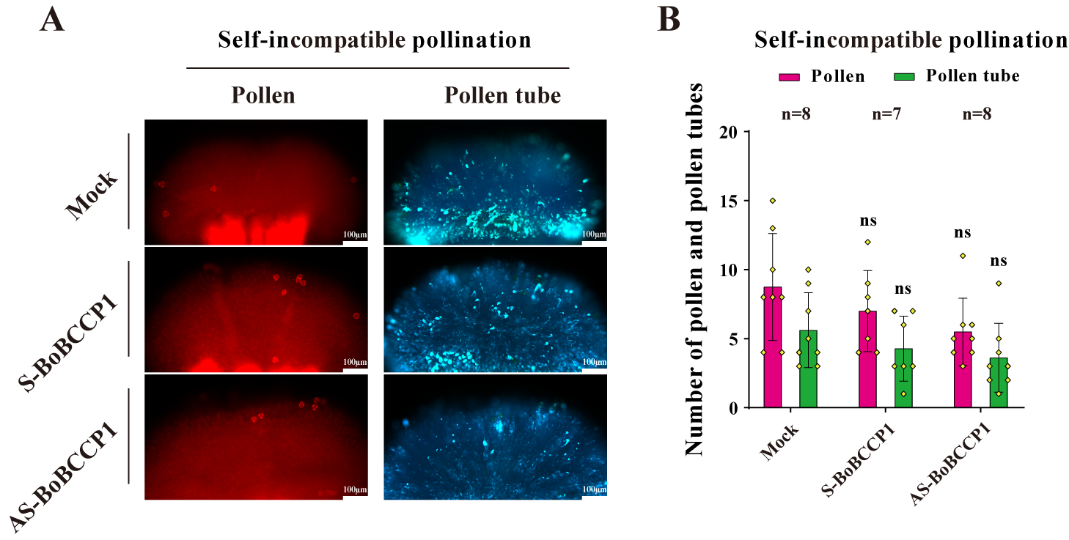


**Supplementary Data Figure S8.** Treatment with AS-BoBCCP1 did not have a significant effect on pollen attachment following self-pollination. **A** Fluorescence microscopy images showing pollen and pollen tube growth on *S_13-b_S_13-b_* stigmas with S-BCCP1 or AS-BCCP1 treatment following self-incompatible pollination. **B** Quantification of pollen attachment and pollen tube growth with S-BCCP1 or AS-BCCP1 treatment following self-incompatible pollination. The data shows no significant changes in pollen attachment or pollen tube growth. Error bars represent the standard error of the mean, and the sample size (n) for each group is provided. Scale bars in the microscopy images represent 100 µm.
